# Efficacy of Dextrose Versus Breast Milk on Pain and Comfort in Neonates Undergoing Heel Lance: A Pilot Study

**DOI:** 10.7759/cureus.89844

**Published:** 2025-08-11

**Authors:** Sabitri Acharya, Pity Koul, Kalpana Sharma

**Affiliations:** 1 Child Health Nursing, Sharda School of Nursing Sciences and Research, Sharda University, Greater Noida, IND; 2 Adult Health Nursing, School of Nursing, Chitwan Medical College, Bharatpur, NPL

**Keywords:** comfort, dextrose, expressed breast milk, heel lance, neonates, pain

## Abstract

Background

Heel-lance procedures are routinely performed in neonates for blood sampling but are known to cause significant pain and distress. Managing procedural pain in newborns is essential not only to ensure immediate comfort but also to promote positive early interactions with healthcare. This study aimed to assess the effectiveness of 25% dextrose and expressed breast milk in reducing pain and enhancing comfort in neonates undergoing heel-lance procedures.

Methods

A quasi-experimental study with pre-test post-test design was undertaken in Lumbini Provincial Hospital and Siddhartha Women and Children Hospital of Lumbini Province, Nepal. A total of 14 neonates admitted to the intensive care unit and meeting the eligibility criteria were taken as the sample of the study. The neonates were assigned to two groups. i.e., intervention group (n=7) who received 2 mL of 25% glucose and control group who received 2ml of breast milk (n=7), two minutes before the heel-lance procedure. Pain and comfort were assessed using the Premature Infant Pain Profile-Revised (PIPP-R) and the Modified Comfort Behaviour Scale (MCBS) at baseline and following the lancing procedure. PIPP-R and MCBS scales were validated with reliability coefficients of 0.76 and 0.82, respectively. The data was analysed descriptively and inferentially using a parametric test.

Results

The mean birth weight of the neonates was 2920 ± 430 grams. Post-intervention analysis showed that the mean PIPP-R score was significantly lower in the 25% dextrose group (3.5 ± 0.78) compared to the expressed breast milk group (8.57 ± 1.51) (P < 0.01), indicating greater pain relief in the dextrose group. Similarly, discomfort levels at 2- and 4-minute post-procedure were significantly reduced in the dextrose group across both sessions (p < 0.01). A strong positive correlation was observed between post-procedural pain and discomfort scores (r = 0.84, p < 0.01), suggesting that higher pain was associated with greater discomfort.

Conclusion

The study findings indicate that 25% dextrose is more effective than expressed breast milk in reducing pain and discomfort in neonates undergoing heel-lance procedures. Incorporating 25% dextrose as a simple, safe, and effective pain management strategy can enhance neonatal comfort during routine clinical interventions.

## Introduction

Neonates in neonatal intensive care units (NICU) endure numerous painful diagnostics or care-related procedures throughout their hospital stays. On average, each neonate in the NICU undergoes between 7.5 and 17.3 painful procedures daily, with the majority of these procedures causing moderate to severe pain [1,2]. Neonates in hospitals undergo 7-17 painful procedures daily, primarily heel lancing, suctioning, and venepuncture, often without pain relief (42%-100%) [3]. In a Jordanian study of 150 neonates, a total of 14,008 painful treatments were recorded, averaging 97.11 procedures per baby and 13.9 per day [4]. Another study conducted in Ethiopia involving 325 neonates found that each infant underwent a median of 4 (ranging from 3 to 7) painful procedures within the first 24 hours of their NICU stay [5]. The most common procedures were heel lancing (20.7%) and venepuncture (18.41%) [5].

Inadequately treated pain during the neonatal period can cause physiological and behavioral changes, potentially leading to long-term issues such as altered pain perception, neurobehavioral changes, and learning disabilities [6]. Acute pain or injury in neonates is a risk factor for permanent neurological damage. The experience of acute and recurrent mild pain can lead to long-term adverse effects on neurological development [7]. Preventing pain and providing effective pain management should be integral components of standard care in the NICU [8]. Although healthcare providers are aware of pain management guidelines for neonates and analgesic strategies, these practices are rarely implemented. Scientific evidence supports the effectiveness of non-pharmacological pain relief methods, such as breastfeeding, skin-to-skin contact, sweet solutions, and multisensory stimulation for managing procedural pain in neonates [9]. Despite this, minor invasive procedures are often performed without the use of pain relief measures [10,11].

While global guidelines such as those from the World Health Organization and the Baby-Friendly Hospital Initiative strongly advocate exclusive breastfeeding and discourage supplemental solutions except when medically necessary, the use of 25% dextrose for procedural pain relief is a well-recognized, clinically justified exception [12]. Administered in minimal volumes, 25% dextrose provides rapid and effective analgesia by activating endogenous opioid pathways via sweet taste receptors in the oral mucosa. Although expressed breast milk offers some analgesic benefits owing to its palatability and bioactive properties, its effect tends to be milder and less consistent compared to 25% dextrose, and its administration depends on maternal availability and the neonate’s feeding readiness [13]. The practicality of 25% dextrose, given its ease of administration, standardized dosing, and prompt action, makes it especially suitable for timely pain management during NICU procedures. Therefore, despite breastfeeding promotion policies, the therapeutic use of 25% dextrose is ethically and clinically appropriate to minimize neonatal pain and discomfort. Hence, the study was undertaken to evaluate the efficacy of 25% dextrose and expressed breast milk in alleviating procedural pain and enhancing comfort, thereby contributing valuable evidence to guide pain management practices that harmonize effective analgesia with breastfeeding support in neonatal care.

## Materials and methods

Research design

The quasi-experimental study with pre-test post-test control group design was undertaken to evaluate the efficacy of dextrose and breast milk on pain and comfort among neonates during a heel lance.

Ethical considerations

Ethical approval was granted by the Ethical Review Board, Nepal Health Research Council, Kathmandu, with approval number 948, dated December 29, 2023. Administrative permissions were granted by Lumbini Provincial Hospital and Siddhartha Children and Women's Hospital. Written informed consent was secured from the neonates’ mothers. Data were collected from February to March 2024. The study was registered with the Clinical Trial Registry of India (CTRI/2024/01/061630).

Study setting and participants

This pilot study was conducted in the neonatal intensive care unit of selected hospitals of Lumbini Province, Nepal, from February 15 to March 10, 2024. A total of 14 neonates were selected based on inclusion criteria into two groups, i.e., intervention groups (25% dextrose group and breast milk group). The sample comprised term neonates up to 28 days old who were admitted to the NICU, required heel-lance procedures, were orally fed with their last feed given at least 30 minutes before the procedure, and were awake. Exclusion criteria included preterm or post-term neonates, those receiving intravenous fluids, or those who were critically ill.

Sample size and sampling technique

The sample size for this study was determined using G*Power statistical software (version 3.1.9.4, Heinrich-Heine-Universität Düsseldorf, Germany) with a significance level of 0.05. The total sample size was determined to be 140. Thus, for the feasibility and validity of the research design, 14 neonates were recruited, which is 10% of the total sample size. 

Purposive sampling technique was adopted to intentionally select neonates who met specific inclusion criteria relevant to the research objectives. This non-probability sampling method enabled the deliberate selection of term neonates admitted to the NICU who were undergoing heel-lance procedures and met specific conditions, including being orally fed and awake before the procedure.

Intervention

Before commencing the study, all nurses working in the NICU were thoroughly trained on the study protocol and the proper administration techniques for both 25% dextrose and expressed breast milk to neonates undergoing heel-lance procedures. Each day, term neonates who met the inclusion criteria were identified using a standardized assessment proforma and were then assigned alternately to either the intervention group (receiving 25% dextrose) or the control group (receiving expressed breast milk) based on their NICU bed serial numbers. The principal investigator, who remained blinded to the group allocations, performed the heel-lance procedures twice daily, once in the early morning and once in the evening, and was responsible for collecting data related to neonatal pain and comfort.

To ensure accurate baseline measurements, neonates were gently awakened from sleep, when necessary, with their level of arousal assessed by observing behaviours such as crying, eye opening, and body movements. Baseline sociodemographic data, as well as pain and comfort levels, were recorded before administering the intervention. The assigned intervention, 2 mL of either 25% dextrose or expressed breast milk, was administered by the trained on-duty nurse using a sterile dropper. Throughout the procedure, neonatal responses were continuously monitored using calibrated standard equipment. Pain was assessed at multiple time points: baseline, and at 30-, 60-, 90-, and 120-seconds post-intervention. Similarly, comfort was assessed at baseline, after two and four minutes following the heel lance, utilizing video recordings reviewed in consultation with a pediatrician.

To maintain the integrity of the study and prevent bias, the on-duty nurses responsible for administering the interventions kept detailed, confidential records of group allocations, which were only shared with the principal investigator after all data collection was completed. This rigorous approach ensured that the investigator remained blinded throughout the study, allowing for objective evaluation of the interventions’ effectiveness.

Assessment of the outcomes

The primary outcome measure was pain, assessed using the Premature Infant Pain Profile-Revised (PIPP-R) [14]. This score includes factors such as maximum heart rate, minimum oxygen saturation levels, gestational age, baseline behavioral state, and the presence of three facial reactions: nasolabial furrow, brow bulge, and eye squeeze [14]. The interclass correlation consistency (ICC) of PIPP-R was 0.79. Pain was assessed at multiple time points: baseline, and at 30-, 60-, 90-, and 120-seconds post-intervention. Similarly, the comfort level of the neonates was assessed by a Modified Comfort Behaviour Scale (MCBS), which was developed by researchers themselves. The tool has been validated based on the literature and researcher discussion. The Cronbach's alpha was 0.82, indicating good reliability. It assesses the indicators like alertness, calmness, crying, duration of crying, muscle tone, and facial tension. Comfort was assessed after two and four minutes of the heel-lance procedure.

Statistical analysis

The data were organized and entered into SPSS Statistics for Windows, Version 16.0 (SPSS Inc., Chicago, IL, USA) for analysis. Continuous variables were presented as mean and standard deviation, and categorical variables as frequencies and percentages. Independent Student’s t-test was used to compare mean values between the two groups. Pearson’s correlation test was used to assess the relationship between pain and comfort. A significance level of p < 0.05 with a 95% confidence interval was considered statistically significant. The significance level was set at P < 0.05, with a 95% confidence interval (CI).

## Results

Table 1 revealed the neonatal characteristics in the intervention and control groups. All neonates were between 0 to 7 days old, with an equal gender distribution of 57.1% male subjects and 42.9% female subjects in both groups. Birth weights in the dextrose group were mostly ≤2500 grams (71.4%) with the remainder between 2501 and 3500 grams (28.5%), whereas all neonates in the breast milk group weighed between 2501 and 3500 grams. Current weights reflected a similar pattern. Importantly, no complications were reported in either group following the intervention, indicating that both treatments were safe and well tolerated.

**Table 1:** Frequency and percentage distribution of neonatal characteristics in intervention and control group

Variables	Intervention group frequency/percentage	Control group frequency/percentage
Days of life		
0-7 days	7 (100)	7 (100)
8-28 days	00	00
Gender		
Male	4 (57.1)	4 (57.1)
Female	3 (42.9)	3 (42.9)
Birth weight		
≤ 2500	5 (71.4)	00
2501 – 3500	2 (28.5)	7 (100)
Apgar		
Severely depressed	00	00
Moderately depressed	2 (28.5)	1 (14.28)
Normal	5 (71.4)	6 (85.71)
Complications after intervention		
Yes	00	00
No	7 (100)	7 (100)

Table 2 presents a comparison of mean pain scores between the intervention group (25% dextrose) and the control group (expressed breast milk) during the first and second heel prick procedures. At baseline, pain scores were identical in both groups (mean = 4.57 ± 0.97), with no statistically significant difference (p = 0.97). However, significant differences emerged following the heel pricks. In both sessions, the intervention group consistently demonstrated significantly lower pain scores at each 30-second interval post-heel prick compared to the control group (p < 0.01). For instance, at 0-30 seconds after the first heel prick, the intervention group had a mean score of 8.71 ± 1.11 versus 14.00 ± 2.51 in the control group. Similarly, by 91-120 seconds in the second heel prick, the intervention group recorded a mean score of 3.5 ± 0.78, substantially lower than 8.57 ± 1.51 in the control group. These findings indicate that 25% dextrose was significantly more effective than expressed breast milk in reducing pain intensity following heel pricks.

**Table 2:** Independent Student’s t-test to compare mean pain scores between intervention and control group Values denote mean ± standard deviation; statistically significant at p < 0.05.

Pain	Intervention group	Control group	p-value
First heel prick			
Baseline	4.57 ± 0.97	4.57 ± 0.97	0.97
0-30 sec	8.71 ± 1.11	14.00 ± 2.51	<0.01
31-60 sec	7.42 ± 1.27	11.57 ± 1.98	<0.01
61-90 sec	5.42 ±1.51	10.14 ± 1.21	<0.01
91-120 sec	4.85 ± 1.34	8.71 ± 1.11	<0.01
Second heel prick			
Baseline	4.57 ± 0.97	4.57 ± 0.97	0.97
0-30 sec	7.57 ± 1.71	13.28 ± 2.81	<0.01
31-60 sec	6.42 ± 1.71	11.71 ± 1.60	<0.01
61-90 sec	4.85 ± 1.34	10.00 ± 1.41	<0.01
91-120 sec	3.5 ± 0.78	8.57 ± 1.51	<0.01

Table 3 presents the mean comfort scores of neonates in both the intervention group (25% dextrose) and the control group (expressed breast milk) during the first and second heel pricks. At baseline, there were no statistically significant differences between the groups for both heel pricks (p > 0.05), indicating similar levels of comfort before intervention. However, post-intervention scores showed significantly lower comfort scores in the intervention group at each time point, indicating better comfort compared to the control group. During the first heel prick, the mean score at 0-2 minutes was 15.85 ± 0.89 in the intervention group, significantly lower than 20.71 ± 1.79 in the control group (p < 0.01). At 2-4 minutes, this difference persisted with scores of 12.42 ± 2.63 and 18.57 ± 1.71, respectively (p < 0.01). A similar pattern was observed in the second heel prick: at 0-2 minutes, the intervention group scored 13.57 ± 1.71 versus 20.14 ± 1.34 in the control group (p < 0.01), and at 2-4 minutes, the intervention group showed significantly greater comfort with a mean score of just 0.71 ± 1.49 compared to 16.71 ± 1.60 in the control group (p < 0.01). These findings suggest that 25% dextrose was more effective in reducing procedural discomfort in neonates than expressed breast milk.

**Table 3:** Independent Student’s t-test to compare mean comfort scores between intervention and control group Values denote mean ± standard deviation; statistically significant at p < 0.05.

Heel prick	Intervention group	Control group	p-value
First heel prick			
Baseline	8.71 ± 1.11	9.42 ± 0.78	0.19
0 -2 min	15.85 ± 0.89	20.71 ± 1.79	<0.01
2 – 4 min	12.42 ± 2.63	18.57 ± 1.71	<0.01
Second heel prick			
Baseline	9.57 ± 0.53	10.00 ± 0.01	0.07
0 -2 min	13.57 ± 1.71	20.14 ± 1.34	<0.01
2 -4 min	0.71 ± 1.49	16.71 ± 1.60	<0.01

Table 4 displays a negative but non-significant relationship between pain and comfort scores during the pre-test phase (r = -0.37, p = 0.18), indicating that higher pain scores were moderately associated with lower comfort, but the association was not statistically significant. In contrast, a strong and statistically significant positive correlation was found in the post-test phase (r = 0.84, p < 0.01), suggesting that as pain scores increased, discomfort levels (indicated by higher comfort scores) also increased. This strong correlation in the post-test indicates a consistent and meaningful relationship between pain and discomfort during and after the heel prick procedure.

**Table 4:** Pearson's 'r' correlation to correlate pain and comfort Statistically significant at p < 0.05.

Pain	Comfort	
	Pearson's ‘r’ value	p-value
Pre-test	-0.37	0.18
Post-test	0.84	<0.01

## Discussion

Blood withdrawal for investigations is a routine procedure in neonates during their hospitalization in NICUs, often resulting in pain and discomfort. Pharmacological measures, such as opioids, are commonly administered for pain management in intensive care settings. However, there is a lack of widespread and effective pain relief interventions for less critically ill neonates. Pain and discomfort in newborns have been shown to affect cardiovascular function, metabolism, and intracranial pressure. Consequently, identifying a simple and acceptable method for enhancing comfort is imperative for both medical and ethical reasons. An ideal analgesic method or drug for neonates should be user-friendly, well-tolerated, minimally invasive, have a rapid onset of action, and exhibit minimal adverse effects.

The study shows that 25% dextrose is more effective in reducing pain and discomfort caused by heel pricks. This is consistent with a study conducted in Gujarat, India, which compared the effects of dextrose and expressed breast milk (EBM) using the PIPP-R scale [15]. The study found that the pain score at 1-1½ minutes post-heel prick in the EBM and 25% dextrose groups was statistically significant (P < 0.003), suggesting the continued analgesic effect of 25% dextrose over EBM [15]. Additionally, another study in Karnataka, India, reported mean pain scores of 3.12, 4.68, and 7.83 at 5-5½ minutes post-heel prick, indicating that 25% dextrose was superior to both breast milk and sterile water [16]. Furthermore, a study in Jharkhand, India, yielded similar results, highlighting that 2 mL of 25% dextrose was more effective in reducing procedural pain from venepuncture compared to 5 mL of EBM [17]. Moreover, physiological markers such as heart rate and oxygen saturation returned to baseline faster and more completely in the 25% dextrose group [17]. The relief in discomfort provided by 25% dextrose or expressed breast milk is likely due to the association between the sweet taste and the activation of the body’s endogenous opioid pathways [18,19]. This sensitivity to sweet taste is well-developed even in all neonates at birth. This sensitivity to sweet taste is well-developed in neonates from birth. A study in Kerala, India, demonstrated mean pain scores of 4.67 ± 1.04 and 6.62 ± 0.82 at the 1st minute, 2.04 ± 1.45 and 4.08 ± 1.05 at the 2nd minute, and 1.63 ± 0.76 and 3.02 ± 1.08 at the 3rd minute for the dextrose and breast milk groups, respectively, with a significant difference (P < 0.001) [20]. The lower mean scores in the dextrose group highlight the importance of non-pharmacological measures in reducing pain from minor invasive procedures compared to breastfeeding [20].

The findings suggest that 25% dextrose is more effective than expressed breast milk in reducing procedural pain in neonates. However, further confirmatory studies are necessary. One trial indicated that oral sucrose did not affect the magnitude of spinal nociceptive reflexes or the acute activation of pain networks in the brain, despite significantly reducing observed pain according to the pain scale. This finding is controversial, especially given that acute pain activates the somatosensory cortex in neonates, and there is substantial evidence that sugar solutions reduce procedural pain in neonates [21]. Notably, the present study included only a small sample size of 14 neonates, underscoring the need for additional studies. Similar research is also needed to explore the effects of expressed breast milk on nociceptive pathways in the neonatal spine and brain. To date, no studies have compared the comfort of neonates during heel-lancing by directly comparing these two interventions. However, other non-pharmacological interventions, like skin-to-skin contact, have been studied in this context. A study in Denmark examined the relationship between skin-to-skin contact and changes in the COMFORTneo score, heart rate, and oxygen saturation during heel-lancing in 67 infants with skin-to-skin contact and 108 infants without [22]. The study found no significant association between skin-to-skin contact and changes in heart rate, oxygen saturation, or the COMFORTneo score from baseline to post-procedure (all, p > 0.05). The study concluded that although skin-to-skin contact offers numerous benefits, there may be situations where its use is not feasible or justified, especially when alternative pain-relieving methods are available and practical constraints exist. Other non-pharmacological approaches should, therefore, be considered to assess the comfort of neonates during minor invasive procedures. Based on the results of previous trials, it can be concluded that small amounts of human milk given two minutes before a painful procedure are not an effective analgesic strategy for neonates. However, it is important not to assume these results are definitive regarding the role of EBM in neonatal analgesia [23,24].

## Conclusions

In conclusion, the present study demonstrates that both 25% dextrose and expressed breast milk are effective in reducing pain and discomfort during heel-lancing in term neonates. However, 25% dextrose showed comparatively greater effectiveness in alleviating pain and enhancing comfort. Owing to its ease of use and longer-lasting analgesic effect, 25% dextrose is recommended as a preferred pain management strategy in clinical settings, particularly in resource-limited countries, to improve neonatal comfort during minor invasive procedures such as heel-lancing.
